# Real-world outcomes and safety of colistin therapy in children with multidrug-resistant gram-negative infections: a nine-year experience

**DOI:** 10.1007/s00431-026-06863-0

**Published:** 2026-03-20

**Authors:** Nesli Ağralı Eröz, Zümrüt Şahbudak Bal, Kübra Cebeci, Gülizar Turan, Nihal Karadaş, Deniz Yılmaz Karapınar, Ezgi Kıran Taşçı, Miray Karakoyun, Gökçen Kartal Öztürk, Gülcihan Özek, Coskun Ekemen, Asli Arslan, Melike Yaşar Duman, Feriha Çilli, Gülhadiye Avcu

**Affiliations:** 1 Division of Pediatric Infectious Diseases, Tepecik Training and Research Hospital, İzmir, Turkey; 2 Division of Pediatric Infectious Diseases, Ege University Faculty of Medicine Hospital, İzmir, Turkey; 3 Division of Pediatric Intensive Care, Ege University Faculty of Medicine Hospital, İzmir, Turkey; 4 Division of Pediatric Hematology, Ege University Faculty of Medicine Hospital, İzmir, Turkey; 5 Division of Pediatric Gastroenterology, Ege University Faculty of Medicine Hospital, İzmir, Turkey; 6 Division of Pediatric Pulmonology, Ege University Faculty of Medicine Hospital, İzmir, Turkey; 7 Bone Marrow Transplantation Unit, Ege University Faculty of Medicine Hospital, İzmir, Turkey; 8 Department of Medical Microbiology, Ege University Faculty of Medicine Hospital, İzmir, Turkey

**Keywords:** Colistin, Antimicrobial resistance, Multidrug-resistant bacteria, Carbapenem-resistant Gram-negative bacteria

## Abstract

Antimicrobial resistance (AMR) among Gram-negative (GN) pathogens has emerged as a critical global threat, particularly in low- and middle-income countries where access to novel antimicrobials is limited. In Türkiye, colistin (CST) remains a last-resort therapy against multidrug-resistant (MDR) and carbapenem-resistant Gram-negative bacteria (CR-GNB) despite its nephrotoxic potential. This study aimed to evaluate the clinical characteristics, microbiological outcomes, and adverse events associated with CST therapy in pediatric patients over a nine-year period. We conducted a single-center retrospective cohort study including pediatric patients treated with CST for nosocomial MDR-GN infections at a tertiary university hospital between January 2016 and March 2025. Demographic, clinical, microbiological, and treatment-related data were collected. Univariable comparisons and multivariable logistic regression analyses were performed to identify factors associated with nephrotoxicity, treatment failure, and mortality. A total of 117 treatment episodes were identified among 112 patients (mean age: 75.9 ± 69.5 months; 65% male). The predominant pathogens were *Klebsiella pneumoniae* (39.7%), *Pseudomonas aeruginosa* (26.4%), and *Acinetobacter baumannii* (22.0%). CST was used in combination regimens in all episodes, most commonly with carbapenems (73.5%). Microbiological eradication was achieved in 70% of culture-confirmed episodes. The overall sepsis-related mortality rate was 18.8%. Nephrotoxicity occurred in 12.8% of patients and was associated with mortality and severity indicators in univariable analyses. In multivariable analysis, endotracheal intubation remained the only independent predictor of mortality (adjusted OR 23.98, 95% CI 4.47–128.52).

*Conclusions*: CST remains a last-resort agent for MDR and CR-GNB infections in pediatric settings with limited access to novel antimicrobials. Although microbiological eradication was achieved in most cases, treatment outcomes were primarily driven by disease severity and host-related factors rather than antimicrobial combinations. Endotracheal intubation emerged as the strongest independent predictor of mortality.

**Table:** 

**What is Known – What is New** •*CST is widely used as a last-resort option for pediatric MDR-GN infections.* •*Nephrotoxicity and variable clinical outcomes remain major concerns.* •*This study provides long-term real-world pediatric data on CST use.* • *Disease severity and host-related factors were the main determinants of treatment failure and mortality.*

**Supplementary Information:**

The online version contains supplementary material available at 10.1007/s00431-026-06863-0.

## Introduction

AMR among GN pathogens has become a major global health concern, contributing substantially to morbidity, mortality, and healthcare costs. Infections caused by MDR and CR-GNB, including *Klebsiella pneumoniae, Acinetobacter baumannii*, and *Pseudomonas aeruginosa*, have reached alarming levels worldwide [1–3]. In 2019, it was estimated that around 1.3 million deaths worldwide were directly linked to AMR pathogens [4]. Although new β-lactam/β-lactamase inhibitor combinations have become available globally, [5–8]. their accessibility in Türkiye is extremely limited, similar to other resource-limited settings. Currently, in our country, ceftazidime–avibactam (CZA-AVI) is the only new-generation β-lactam/β-lactamase inhibitor combination approved for clinical use against certain carbapenem-resistant Enterobacterales (CRE); however, its high cost and restricted availability have limited its widespread use in daily practice. Consequently, CST remains a cornerstone of treatment for resistant GN infections at many centers across the country.

CST is a cationic polypeptide antibiotic that was reintroduced into clinical practice as a last-resort therapy against MDR-GN bacteria after decades of disuse due to concerns about nephrotoxicity and neurotoxicity [9]. Despite these adverse effects, CST retains potent activity against CR organisms and continues to be used widely, particularly in intensive care units and immunocompromised patients. Nevertheless, data on its clinical efficacy, microbiological eradication rates, and safety profile remain limited, especially in pediatric patients, and reported outcomes vary with infection severity, dosing strategies, and concomitant nephrotoxic agents [10–12]. There is limited pediatric real-world data evaluating not only microbiological outcomes but also host-related predictors of failure and nephrotoxicity during prolonged CST exposure.

In this study, we aimed to evaluate the clinical characteristics, microbiological profiles, treatment outcomes, and adverse events among pediatric patients who received CST therapy for resistant GN infections over nine years at a tertiary-level university hospital. Our findings provide real-world evidence on the role of CST in treating MDR and CR-GNB infections, particularly in settings with limited access to novel antimicrobials.

## Materials and methods

This retrospective study was conducted at Ege University Children’s Hospital, a tertiary-level university hospital. The study included pediatric patients who received CST therapy, either empirically or as targeted therapy for suspected or culture-confirmed MDR nosocomial infections, between January 2016 and March 2025. Data were collected from electronic medical records. The following parameters were reviewed: demographic information, underlying diseases, comorbidities, isolated pathogens and their antimicrobial susceptibility profiles, use of concomitant nephrotoxic agents, hospital and PICU length of stay, and the requirement for mechanical ventilation (MV) and inotropic support. The presence of invasive medical devices was also recorded. Detailed data on infection characteristics were collected, encompassing infection type and site [e.g., bloodstream infection (BSI), ventilator-associated pneumonia (VAP), meningitis, or others]. Therapeutic variables—including the route of CST administration, treatment duration, and the occurrence of treatment-related adverse events—were systematically documented. Clinical and laboratory parameters were evaluated, including serum urea and creatinine levels (SCr), C-reactive protein (CRP), leukocyte and platelet counts, and procalcitonin (PCT) levels when available. The occurrence of nephrotoxicity, neurotoxicity (manifested as seizures, altered consciousness, or neuromuscular blockade), and hypersensitivity reactions related to CST therapy were systematically recorded. For nephrotoxicity assessment, SCr levels measured immediately before CST initiation and the highest SCr value recorded during therapy were used for comparison. Nephrotoxicity was defined according to the Kidney Disease: Improving Global Outcomes (KDIGO) criteria, which include either an increase in SCr of ≥ 0.3 mg/dL (≥ 26.5 μmol/L) within 48 h or an increase of ≥ 50% from baseline within the previous 7 days [13]. Concomitant use of nephrotoxic agents, including amphotericin B (AmB), aminoglycosides, cyclosporine, acyclovir, ganciclovir, vancomycin, intravenous contrast agents, and chemotherapeutic drugs, was also documented to identify potential risk factors for renal impairment.

Treatment outcomes were classified based on clinical and microbiological responses and overall prognosis.

A new infection episode was defined as a microbiologically documented infection occurring after completion of appropriate antimicrobial therapy for the preceding episode, with resolution of clinical signs and symptoms of infection and at least one sterile culture from the previously infected site. Subsequent infections occurring before clinical and microbiological resolution were classified as belonging to the same episode.

Patients who were neonates or who received CST therapy for fewer than 6 doses or for fewer than 48 h were excluded. Patients with incomplete or missing clinical, microbiological, or treatment-related data were excluded from the analysis.

### Colistin administration and dosing

CST therapy was administered via different routes. Intravenous CST was administered at daily doses ranging from 2.5 to 5 mg colistin base activity (CBA)/kg. Inhaled CST was administered as adjunctive therapy at a dose of 150 mg CBA per administration. Intrathecal CST was administered at a dose of 10 mg colistimethate sodium per day; in all such cases, intravenous CST was administered concomitantly. Intravenous CST therapy administered for more than 48 h was defined as continuous treatment**.**

### Microbiological testing

Presumptive identification of GN pathogens was performed using the VITEK MS system (bioMérieux, France). This system uses matrix-assisted laser desorption/ionization time-of-flight mass spectrometry (MALDI-TOF MS), a modern technology that enables rapid, accurate species identification based on the unique protein composition of microbial cells.

Antimicrobial susceptibility testing was carried out on Mueller–Hinton agar using the Kirby–Bauer disk diffusion method in accordance with standard protocols. The susceptibility of isolates to amikacin (AMK), ceftriaxone (CRO), CZA, piperacillin–tazobactam (TZP), cefoperazone–sulbactam (CFP-SUL), imipenem (IPM), meropenem (MEM), CST, and tigecycline (TGC) was determined following the recommendations of the European Committee on Antimicrobial Susceptibility Testing (EUCAST) [14].

Since the broth microdilution method for CST susceptibility testing became available only after August 2023, CST minimum inhibitory concentration (MIC) values and susceptibility results were evaluated exclusively for isolates tested after this date to ensure accuracy and standardization.

### Definitions

We retrospectively reviewed the medical records of all eligible patients and collected comprehensive demographic, clinical, and microbiological data using a standardized pediatric infectious diseases consultation form. Both data collection and treatment decisions were performed under the supervision of a pediatric infectious diseases specialist and the pediatric infectious diseases team. Epidemiological parameters included age, sex, underlying medical conditions (such as pulmonary, cardiovascular, renal, hepatic, or metabolic diseases, malignancy, hematologic or solid organ transplantation, genetic syndromes), and treatment-related factors such as the presence of a central venous catheter (CVC), foley catheter, MV, and prior antibiotic exposure. Microbiological cultures were obtained based on the attending physician's clinical suspicion. The diagnosis of infection was established according to clinical findings and the isolation of pathogens from normally sterile sites.

Nosocomial infections were defined according to the CDC National Healthcare Safety Network (NHSN) criteria: infections that developed ≥ 48 h after hospital admission in patients without evidence of active infection at entry and that met CDC clinical and laboratory diagnostic thresholds. Hospital-acquired pneumonia (HAP) was defined according to the Infectious Diseases Society of America/American Thoracic Society (IDSA/ATS) guidelines as pneumonia developing ≥ 48 h after hospital admission, characterized by a new or progressive radiographic infiltrate plus at least one clinical feature of infection—fever, leukocytosis or leukopenia, or purulent respiratory secretions—supported by compatible respiratory signs [15].

Ventilator-associated pneumonia (VAP) was defined according to CDC/NHSN surveillance criteria, requiring worsening oxygenation, a new or progressive radiographic infiltrate, and clinical signs of infection, including fever, leukocytosis or leukopenia, and purulent endotracheal secretions, occurring after ≥ 48 h of MV [16].

Central line–associated bloodstream infections (CLABSI) were defined according to CDC/NHSN standardized criteria as laboratory-confirmed bloodstream infections occurring in patients with a CVC in place for more than 48 h, with no alternative source of infection identified [17].

Sepsis was defined according to the International Pediatric Sepsis Consensus definitions, requiring systemic inflammatory response syndrome criteria in the context of suspected or confirmed infection, accompanied by age-adjusted physiologic abnormalities [18].

CRE are defined as members of the Enterobacterales order resistant to at least 1 carbapenem antibiotic or producing a carbapenemase enzyme [19].

MDR *P. aeruginosa* is defined as *P. aeruginosa* not susceptible to at least 1 antibiotic in at least 3 antibiotic classes for which *P. aeruginosa* susceptibility is generally expected: penicillins, cephalosporins, fluoroquinolones, aminoglycosides, and carbapenems [20].

Difficult-to-treat resistant (DTR) is defined as *P. aeruginosa* exhibiting non-susceptibility to all of the following: TZP, CZA, cefepime (FEP), aztreonam, MEM, IPM, ciprofloxacin (CIP), and levofloxacin (LVX) [21].

Clinical response was defined as the complete resolution of infection-related symptoms and signs, whereas microbiological response was defined as culture negativity at the end of treatment. Treatment failure was defined as persistent or worsening clinical or radiological findings or continued pathogen isolation despite therapy. Superinfection was defined as a new infection caused by another organism occurring at least 72 h after CST initiation.

### Ethical considerations

The study was approved by the Research Ethics Committee of Ege University Faculty of Medicine and by the Turkish Ministry of Health (Approval No: 25-5 T/91). All study procedures were conducted in accordance with the principles outlined in the Declaration of Helsinki.

### Statistical analysis

All statistical analyses were performed using SPSS Statistics software, version 27.0 for Windows (IBM Corp., Armonk, NY, USA). Continuous variables were expressed as mean ± standard deviation (SD) for normally distributed data or as median (interquartile range) for non-normally distributed data, while categorical variables were presented as frequencies and percentages.

Comparisons between groups were performed using the Student’s t-test for normally distributed continuous variables and the Mann–Whitney U test for non-parametric data. The Chi-square (χ^2^) test or Fisher’s exact test was used, as appropriate, to evaluate associations between categorical variables. Correlation analyses were conducted using Pearson’s correlation coefficient (r).

To identify independent factors associated with treatment outcomes and nephrotoxicity, univariable and multivariable logistic regression analyses were performed. Variables with a p-value < 0.1 in univariable analysis were included in the multivariable logistic regression model. A p-value < 0.05 was considered statistically significant for all tests.

## Results

A total of 117 CST treatment episodes were analyzed in 112 patients who received CST therapy empirically or as targeted treatment for resistant GN infections between January 2016 and March 2025; five patients received CST therapy during two separate infection episodes. Most patients (n = 112, 95%) received intravenous CST at a daily dose of 5 mg CBA/kg**.** Two patients (1.7%) received 4 mg CBA/kg, and three patients (2.5%) received 2.5 mg CBA/kg intravenously. Inhaled CST was used as adjunctive therapy in 9 patients (7.6%).

Intrathecal CST was administered in 3 patients (2.5%)**,** all of whom also received concomitant intravenous CST.

The mean age of the patients was 75.9 ± 69.5 months, and 65% (n = 76) of them were male. Among the patients, 2.5% (n = 3) were healthy with no known underlying disease. The most common comorbidity was malignancy, observed in 26.3% (n = 31) of patients. Other chronic conditions included hematologic or solid organ transplantation in 11% (n = 13), cardiovascular diseases in 10.2% (n = 12) genetic syndromes in 4.2% (n = 5), neurological diseases in 3.4% (n = 4), renal diseases in 3.4% (n = 4), chronic pulmonary diseases in 7.6% (n = 9), metabolic disorders in 2.5% (n = 3), and primary immunodeficiency in 0.8% (n = 1). Other comorbidities were grouped under “others,” comprising 27.1% (n = 32) of the cohort. A total of 64.1% of patients (n = 75) had a prior hospitalization, with a median duration of 7 days (range: 1–139).

CST was administered for nosocomial infections in 96.4% of patients (n = 112), most frequently for sepsis (n = 27; 23.1%), followed by pneumonia (n = 21; 17.9%) and CLABSI (n = 19; 16.2%) (Table 1). A microbiological pathogen was identified in 75% (n = 88) of the patients, with CRE being the most common, detected in 42% (n = 37) of cases (Table 2). Among 88 patients with a pathogen identified, the most common isolate was Klebsiella pneumoniae (n = 35; 39.7%), of which the vast majority (94.5%) were CR. This was followed by *Pseudomonas aeruginosa* (n = 23, 26.1%) and *Acinetobacter baumannii* (n = 21; 23.8%). Other less frequently isolated organisms included *Achromobacter xylosoxidans* (n = 3; 3.4%), *Escherichia coli* (n = 3; 3.4%), and *Elizabethkingia* (n = 1; 1.1%). The resistance rates to AMK, gentamicin, CZA, FEP, CIP, IPM, MEM, and trimethoprim-sulfamethoxazole (TMP-SMX) were 53.5%, 74.4%, 90.7%, 95.2%, 82.3%, 91.0%, 86.0%, and 36.5%, respectively. Since the microdilution method for CST susceptibility testing became available only after August 2023, CST resistance could be evaluated in 11 patients, and no resistance was detected in any of them. CST therapy was initiated as targeted treatment in 70.9% (n = 83) of patients and as empirical therapy in 29.1% (n = 34). The mean duration of antibiotic therapy was 14.7 ± 9.6 days (Table 3).

**Table 1:** Characteristics of the hospitalized patients treated with colistin

Demographics of the treatment episodes (n = 117)	n (%)
Age (mean, standard deviation)	75.9 ± 69.5 months
Sex, male	76 (65)
Comorbidities	114 (97.5)
Malignancy	31 (26.3)
Hematologic/solid organ transplantation	13 (11)
Cardiovascular diseases	12 (10.2)
Genetic syndromes	5 (4.2)
Neurological diseases	4 (3.4)
Renal diseases	4 (3.4)
Chronic pulmonary diseases	9 (7.6)
Metabolic disorders	3 (2.5)
Primary immunodeficiency	1 (0.8)
Others	32 (27.1)
Predisposing factors	
Device	103(88)
Catheter	57 (48.7)
Port catheter	24 (20.5)
EVD	9 (7.7)
Assist device	4 (3.4)
VP shunt	3 (2.6)
Double-J catheter	(2.6)
Pigtail catheter	3 (2.6)
Chest tube	2 (1.7)
Epidural catheter	1 (0.9)
Nephrostomy catheter	1 (0.9%)
PICU admission	71(60,7)
Length of stay at PICU (day)- median(range)	16(1–274)
Total parenteral nutrition	62(53)
Invasive surgical procedures	76(65)
Mechanical ventilation	36(30,8)
Infection	
Nosocomial	112(96.4)
Community acquired	5(3.6)
Type of infection	
Sepsis	27(23.1)
HAP	21(17.9)
CLABSI	19(16.2)
VAP	10(8.5)
UTI	10(8.5)
BSI	8(6.8)
Intra-abdominal infections	8(6.8)
CNS infection	7(6)
Surgical site infection	5(4.3)
Soft tissue infections	1(0.9)
Febrile neutropenia	1(0.9)
Laboratory parameters (mean ± SD)	
WBC (cells/µL)	12,234 ± 16,840
ANC (cells/µL)	5210 ± 8017
Platelet count (10^3/µL)	274 ± 403
CRP, mg/L	88.10 ± 84.16
PCT, µg/L	4.9 ± 14.3
Creatinine (mg/dL)	0.37 ± 0.37
Peak creatinine during colistin therapy (mg/dL)	0.49 ± 0.54

*ANC* Absolute neutrophil count, *BSI* Bloodstream infection, *CLABSI* Central Line-associated Bloodstream Infection, *CNS* Central nervous system, *CRP* C-reactive protein, *EVD* Extraventricular drainage, *HAP* hospital-acquired pneumonia, *PCT* procalcitonin, *PICU* pediatric intensive care unit, *PLT* platelet count, *UTI* Urinary tract infection, *VAP* ventilator-associated pneumonia, *VP* Ventriculoperitoneal, *WBC* white blood cell

**Table 2:** Microorganisms isolated from different sites

Isolated microorganism	Blood[n (%)]	Urine[n (%)]	Endotracheal lavage [n (%)]	Cerebrospinal Fluid [n (%)]	Other [n (%)]	Total[n(%)]
CRE	22 (70.9)	7 (77.7)	2 (8)	1 (20)	5 (29.4)	37 (42)
-*Klebsiella pneumoniae*	22(70.9)	4(44.4)	1(4)	1(20)	5 (29.5)	33(37.9)
-*Escherichia coli*	-	2 (22.2)	1 (4)	-	-	3 (3.4)
*-Klebsiella oxytoca*	-	1 (11.1)	-	-	-	1 (1.1)
CRAB	4 (12.9)	1 (11.1)	6 (24)	2 (40)	7 (41.1)	21 (23.8)
*Pseudomonas aeruginosa*	3 (9.6)	1 (11.1)	14 (56)	1 (20)	4 (23.5)	23 (26.4)
*-*MDR *Pseudomonas aeruginosa*	3 (9.6)	**-**	8 (32)	-	3 (17.6)	14 (16)
*-DTR Pseudomonas aeruginosa*	-	1 (11.1)	6 (24)	1 (20)	1 (9)	9 (10)
ESBL-positive	2 (6.4)	-	-	1 (20)	-	3 (3.4)
*-Klebsiella pneumoniae*	2 (6.4)	-	-	-	-	2 (2.2)
*-Enterobacter* spp	-	-	-	1 (20)	-	1 (1.1)
*Achromobacter xylosoxidans*	-	-	2 (8)	-	1 (9)	3 (3.4)
*Elizabethkingia*	-	-	1 (4)	-	-	1 (1.1)
Total	31	9	25	5	17	88 (100)

*CRAB* carbapenem-resistant Acinetobacter baumannii, *CRE* carbapenem-resistant Enterobacterales, *DTR* difficult-to-treat resistance, *ESBL* extended-spectrum beta-lactamase, *MDR* multidrug-resistant

**Table 3:** Summary of colistin treatment

	Number of patients(%)
Duration of colistin treatment (days)-mean (SD)	14.7 ± 9.6
Colistin Usage	
-Targeted	83(70.9)
-Empirical	34(29.1)
Combined treatment	117(100)
-Combination with a single antibiotic	41(35.3)
-Combinations with multiple antibiotics	76(64.9)
Antibiotics combined	
-Carbapenem	86 (73.5)
-Aminoglycosides	38(32.4)
-Tigecycline	15 (12.8)
-Trimethoprim–Sulfamethoxazole	9 (7.6)
-Fluoroquinolone	15 (12.8)
-Ceftazidime	5 (4.2)
-Ampicillin/Sulbactam	5 (4.2)
-Cefoperazone/Sulbactam	6 (5.1)
-Cefepime	1 (0.9)
-Piperacillin–Tazobactam	1 (0.9)
Colistin sensitivity (n = 11)	
MIC = 1 μg/mL	5 (45)
MIC < 1 μg/mL	3 (27)
MIC = 2 μg/mL	1 (9)
Total adverse events	16 (13.6)
– Nephrotoxicity	15 (12.8)
– Urticaria	1 (0.8)
Nephrotoxicity characteristics	
– Mean time to onset, days (± SD)	5.5 ± 4.8
– Required dialysis	3 (20.0)
– Concomitant nephrotoxic agent use	10 (66.7); p = 0.58
– Non-resolving nephrotoxicity (fatal cases)	7 (46.7)
Concomitant nephrotoxic agents	
– Glycopeptides	3 (20.0)
– Aminoglycosides	3 (20.0)
– Amphotericin B	2 (13.3)
– Ganciclovir	1 (6.7)
– Glycopeptide + Aminoglycoside	1 (6.7)
Outcome	
– Clinical response	72 (62.1)
– Microbiological response	61 (70.2)
– Treatment failure	44 (37.6)

*MIC* minimum inhibitory concentration

The median length of hospital stay was 71 days (range: 6–696), and the median PICU stay was 16 days (range: 1–274). MV was required in 30.8% of patients (n = 36). The median duration of MV was 9 days (range: 1–129). Inotropic support was needed in 34% of patients (n = 40).

### CST Treatment outcomes

CST was administered as combination therapy in all patients. Among the combination regimens, single-antibiotic combinations were applied in 41 (35.3%) patients, and multiple-antibiotic combinations in 76 (64.9%)**.** The most frequently combined antibiotics were carbapenems (n: 86, 73.5%), followed by aminoglycosides (n: 38, 32.4%), TGC (n: 15, 12.8%), fluoroquinolones (n: 15, 12.8%), and TMP-SMX (n: 9, 7.6%)**.** Less commonly used agents included CFP-SUL (n = 6, 5.1%), CZA (n = 5, 4.2%), ampicillin/sulbactam (n = 5, 4.2%), FEP (n = 1, 0.9%), and TZP (n = 1, 0.9%)**.** CST susceptibility testing (available in 11 isolates) showed that 5 isolates (45%) had a MIC of 1 μg/mL**,** 3 isolates (27%) had MIC < 1 μg/mL**,** and 1 isolate (9%) had MIC = 2 μg/mL (Table 3).

Clinical response rates varied by infection type (Table 4). The highest clinical response was observed in HAP (47.6%), followed by BSI and VAP, whereas lower response rates were noted in CLABSI and UTI. The lowest clinical response was observed in patients with surgical site infections (20%). In some cases, microbiological eradication was achieved without concurrent clinical improvement, reflecting persistent systemic inflammation, organ dysfunction, or complications unrelated to ongoing bacterial infection. In addition, clinical response rates varied within the same infection site depending on the causative pathogen. For example, in HAP, higher clinical response rates were observed in infections caused by MDR or DTR *Pseudomonas aeruginosa* than in those caused by carbapenem-resistant Acinetobacter baumannii (CRAB), highlighting that both infection site and pathogen type influence clinical outcomes.

**Table 4:** Microorganisms isolated, treatment type, and clinical response of colistin therapy

Infection Type	Microorganisms n (%)	Clinical Response (n, %)	Microbiological Eradication (n, %)
CLABSI (n = 19)	CRE: 14 (73.7)	4 (28)	12 (85)
	CRAB: 3 (15.8)	1 (33)	3 (66)
	ESBL + *Klebsiella pneumoniae*: 1 (5.3)	-	1 (100)
	MDR *P. aeruginosa*: 1 (5.3)	1 (100)	1 (100)
BSI (n = 8)	CRE: 5 (62.5)	2 (40)	3 (60)
	ESBL + *Klebsiella pneumoniae:* 1 (12.5)	-	1 (100)
	*MDR P. aeruginosa*: 2 (25)	1 (50)	1 (50)
VAP (n = 10)	CRAB: 3 (33.3)	2 (66.6)	2 (66)
	DTR *P. aeruginosa*: 2 (22.2)	-	1 (50)
	CRE: 2 (22.2)	-	-
	MDR *P. Aeruginosa:* 1 (10)	-	1 (100)
	*Achromobacter xylosoxidans:* 1 (10)	1 (100)	1 (100)
	Causative agent not detected: 1 (10)	1 (100)	-
HAP (n = 21)	MDR *P. aeruginosa*: 9 (42.9)	5 (55.5)	3 (33.3)
	DTR *P. aeruginosa*: 5 (23.8)	3 (60)	1 (20)
	CRAB: 4 (19.0)	1 (25)	3 (75)
	*Achromobacter xylosoxidans:* 2 (9.5)	1 (50)	1 (50)
	*Elizabethkingia:* 1 (4.7)	-	1 (100)
CNS infection (n = 7)	CRAB: 2 (28.5)	-	2 (100)
	CRE: 1 (14.2)	-	1 (100)
	DTR *P. aeruginosa:* 1 (14.2)	-	1 (100)
	ESBL + *Enterobacteriaceae*: 1 (14.2)	-	1 (100)
	Causative agent not detected: 2 (28.5)	-	-
UTI (n = 10)	CRE: 7 (77.8)	2 (28.5)	7 (100)
	CRAB: 1 (11.1)	-	1(100)
	DTR *P. aeruginosa*: 1 (11.1)	-	1 (100)
	Causative agent not detected: 1 (11.1)	-	-
Surgical site infection (n = 5)	CRAB: 4 (80)	1 (25)	2 (50)
	Causative agent not detected: 1 (20)	-	-
Intra-abdominal infection (n = 8)	CRE: 4 (50)	1 (25)	2 (50)
	CRAB: 4 (50.0)	2 (50)	3 (75)
Sepsis (n = 27)	CRE: 3 (11.1)	2 (66.6)	2 (66.6)
	MDR *P. aeruginosa*: 1 (3.7)	1 (100)	-
	Causative agent not detected: 23 (85)	11 (47.8)	-
Soft tissue infection (n = 1)	CRE: 1 (100)	1 (100)	1 (100)
Febrile neutropenia (n = 1)	-	-	-

*BSI* Bloodstream Infections, *CLABSI* Central Line-associated Bloodstream Infection, *CNS* central nervous system, *CRAB* Carbapenem-resistant *A. Baumannii*, *CRE* Carbapenem-resistant Enterobacterales, *DTR* Difficult-to-treat resistant *Pseudomonas aeruginosa*, *HAP* Hospital-acquired pneumonia, *MDR* multidrug-resistant, *UTI* urinary tract infection, *VAP* Ventilator-associated pneumonia

### Microbiological results

Microbiological eradication was achieved in 70% (n = 61) of patients with identified pathogens, with a median time to eradication of 4 days (range, 1–33 days). Superinfection occurred in 29% (n = 35) of patients and was most commonly caused by fungal pathogens (n = 8; 22%), including *Candida albicans* (n = 5), *Candida tropicalis* (n = 1), *Candida parapsilosis* (n = 1), and *Pneumocystis jirovecii* (n = 1).

Microbiological eradication was most frequently achieved in UTI (n:9; 100%) and central nervous system (CNS) infections (n:6; 100%), whereas lower eradication rates were observed in sepsis (n:2; 66.7%) and intra-abdominal infections (n:5; 62.5%).

Overall, infections caused by CRAB and DTR *Pseudomonas aeruginosa* were associated with lower clinical and microbiological response rates compared to those caused by CRE (Table 4).

### Adverse events

Nephrotoxicity developed in 12.8% of patients (n = 15), while urticaria related to CST was observed in only 1 patient; no cases of neurotoxicity were observed during the study period (Table 3). Among the 15 patients who developed nephrotoxicity, 10 had concomitant exposure to other nephrotoxic agents; however, no significant association was observed between concomitant nephrotoxic drug use and nephrotoxicity (p = 0.58) (Table 3).

The concomitant nephrotoxic agents included glycopeptides in 3 patients, aminoglycosides in 3, amphotericin B in 2, ganciclovir in 1, and a combination of a glycopeptide and an aminoglycoside in 1 patient. Nephrotoxicity developed at a mean of 5.5 days (± 4.8 SD). Among patients who developed nephrotoxicity, 3 required dialysis (20%). All three patients who required dialysis had concomitant exposure to other nephrotoxic agents: one received ganciclovir, one an aminoglycoside, and one amphotericin B. Nephrotoxicity did not resolve in seven patients, all of whom died during follow-up. Of these seven patients, microbiological eradication could not be achieved in five, whereas in the remaining two, no causative pathogen was identified; both groups ultimately experienced sepsis-related mortality.

Mortality was observed in 18.8% (n = 22) of patients, and it was significantly higher among those who developed nephrotoxicity (n = 7; 31.8%) (p = 0.008). The proportion of female patients was significantly higher in the nephrotoxicity group compared with those without nephrotoxicity (p = 0.04). A history of prior hospitalization was also more common among patients who developed nephrotoxicity (p = 0.01). Similarly, inotropic support (p = 0.03**)** and intubation (p = 0.002) were significantly more frequent in the nephrotoxicity-positive group. Microbiological eradication was achieved less frequently in patients with nephrotoxicity (n = 4; 6.8%) (p = 0.01). In multivariable logistic regression analysis evaluating factors associated with nephrotoxicity, age, PICU admission, sepsis, use of multiple antibiotic combinations, concomitant nephrotoxic medications, and duration of antibiotic therapy were not independently associated with nephrotoxicity (all p > 0.05) (Table 5). The limited number of nephrotoxicity events may have reduced the power to detect independent associations in multivariable analyses.

**Table 5:** Multivariable logistic regression analysis of factors associated with nephrotoxicity

Variable	OR (95% CI)	P value
Age (per month)	1.01 (0.99–1.01)	0.220
PICU admission	1.77 (0.49–6.49)	0.387
Sepsis	0.39 (0.10–1.49)	0.167
Multiple antibiotic combination therapy	0.67 (0.17–2.60)	0.559
Concomitant nephrotoxic therapy	1.07 (0.30–3.79)	0.921
Duration of antibiotic therapy (per day)	0.95 (0.88–1.03)	0.223

*PICU* pediatric intensive care unit

### Outcomes

A total of 41 patients (35%) received CST-based dual combination therapy, and 76 patients (64.9%) received CST-based multiple combination therapy.

The need for inotropic support was significantly higher in the multiple combination group compared with the dual combination group (42%, n = 32 vs. 19%, n = 8; p = 0.01).

Similarly, the incidence of sepsis was significantly greater in patients receiving multiple combinations (61%, n = 46 vs. 31%, n = 13, p = 0.003). Intubation was also more frequent in the multiple therapy group (37%, n = 28 vs. 19%, n = 8; p = 0.05). However, there were no statistically significant differences between the two groups in terms of mortality (21%, n = 16 vs. 14%, n = 6; p = 0.46)**,** nephrotoxicity (14%,n = 11 vs. 9%,n = 4; p = 0.56)**,** clinical response (57%,n = 43 vs. 68%,n = 28; p = 0.32)**,** microbiological eradication (42% vs. 63%, p = 0.89)**,** or treatment failure (42%,n = 32 vs. 29%,n = 26; p = 0.16)**.**

Clinical response was achieved in 55.9% (n = 19) of patients in the empirical therapy group and in 64.6% (n = 53) of those in the targeted therapy group. Although the clinical response rate appeared slightly higher in the targeted therapy group, this difference was not statistically significant (p = 0.40). Similarly, microbiological eradication rates were comparable between the empirical and targeted therapy groups (67%, n = 4 vs. 71%, n = 55; p = 0.58). However, the empirical therapy group included only six patients. The incidence of sepsis was significantly higher in the empirical CST group (n = 22, 64.7%; p = 0.038). While the mortality rate was slightly higher in the empirical group compared to the targeted therapy group (23.5%; n = 8 vs. 16.9%; n = 14), the difference was not statistically significant (p = 0.43).

In univariable logistic regression analysis, increasing age was significantly associated with treatment failure (OR = 1.006, 95% CI: 1.001–1.012, p = 0.024). Admission to the pediatric intensive care unit was associated with a higher likelihood of treatment failure (OR = 4.00, 95% CI: 1.68–9.49, p = 0.002). Neutropenia was significantly associated with increased odds of treatment failure (OR = 3.89, 95% CI: 1.41–10.73, p = 0.009). Nephrotoxicity was associated with treatment failure in a borderline manner (OR = 2.87, 95% CI: 0.95–8.72, p = 0.063). In addition, inotropic support requirement, intubation, and sepsis were strongly associated with treatment failure, with odds ratios of 8.90 (95% CI: 3.72–21.28), 5.06 (95% CI: 2.18–11.74), and 4.54 (95% CI: 2.01–10.28), respectively (all p < 0.001). Sex, device use, and superinfection were not significantly associated with treatment failure (Table 6). Sepsis-related mortality in the overall population was observed in 18.8% (n = 22) of patients.

**Table 6:** Univariable logistic regression analysis of factors associated with treatment failure

Variable	OR	95% CI	P value
Age (months)	1.006	1.001–1.012	0.024
Sex	0.91	0.42–1.99	0.816
PICU admission	4.00	1.68–9.49	0.002
Device use	0.56	0.18–1.72	0.312
Neutropenia	3.89	1.41–10.73	0.009
Nephrotoxicity	2.87	0.95–8.72	0.063
Superinfection	0.97	0.43–2.20	0.946
Inotropic support	8.90	3.72–21.28	< 0.001
Intubation	5.06	2.18–11.74	< 0.001
Sepsis	4.54	2.01–10.28	< 0.001

*PICU* pediatric intensive care unit

A comparison between survivors and non-survivors is presented in Table 7. Non-survivors were significantly more likely to require PICU admission, mechanical ventilation, and had higher rates of sepsis and nephrotoxicity. In addition, microbiological eradication was achieved less frequently among non-survivors (Table 7).

**Table 7:** Comparison of survivors and non-survivors

Variable	Survivors (n = 95)	Non-survivors (n = 22)	p value
Age (months), median (IQR)	48 (10–144)	72 (20.5–147)	0.12
PICU admission, n (%)	52 (54.7)	19 (86.4)	0.006
Mechanical ventilation, n (%)	17 (17.9)	19 (86.4)	< 0.001
Sepsis, n (%)	37 (38.9)	22 (100)	< 0.001
Nephrotoxicity, n (%)	8 (8.4)	7 (31.8)	0.003
Microbiological eradication, n (%)	54 (79.4)	5 (31.3)	< 0.001
Malignancy, n (%)	21 (22.1)	10 (45.5)	0.16

In multivariable logistic regression analysis for mortality, only endotracheal intubation remained independently associated with death (adjusted OR 23.98, 95% CI 4.47–128.52). Nephrotoxicity and microbiological eradication were not independently associated with mortality.

## Discussion

In the context of GN pathogens, the predominance of CRE in our series is consistent with global challenges in AMR. The comparatively lower eradication and clinical response rates observed in infections caused by CRAB and DTR *Pseudomonas aeruginosa* align with prior work indicating these organisms are associated with worse outcomes despite CST therapy [22].

The relatively high microbiological eradication rate observed in our cohort is consistent with previous pediatric reports. These favorable outcomes are consistent with the literature, which reports microbiological clearance rates of 60–80% in children with CST-treated infections [23]. Despite the high prevalence of CRE, CRAB, and other MDR GN pathogens across nearly all infection types, the study demonstrates relatively favorable microbiological outcomes**.** Although CRE and CRAB infections showed high microbiological eradication rates, clinical response varied substantially across infection sites. While infections such as UTIs and some VAP cases demonstrated relatively favorable clinical improvement, outcomes were less favorable in more complex infections such as CLABSI, HAP, and sepsis. These findings indicate that, despite effective microbiological clearance, clinical recovery is strongly influenced by infection site and underlying patient factors**.**

Turning to safety, nephrotoxicity remains the most frequently cited adverse event associated with CST use. In adult studies, rates of nephrotoxicity range widely from 6 to 48%, reflecting heterogeneity in patient populations, dosing strategies, and definitions of renal injury [24]. In our pediatric series, the incidence of nephrotoxicity was 12.8%, which is toward the lower end of that spectrum. However, the association of nephrotoxicity with increased mortality, prior hospitalization, intubation, and the absence of microbiological eradication indicates that renal injury remains a key prognostic marker in CST-treated patients. Although nephrotoxicity was observed in 12.8% of patients and was associated with increased mortality in univariable analyses, it did not remain an independent predictor in the multivariable model. This finding suggests that renal impairment in this cohort may not solely represent a direct adverse drug reaction to CST but may also reflect underlying disease severity, critical illness, multiorgan dysfunction, and prolonged intensive care exposure. In addition, the frequent use of concomitant nephrotoxic agents in critically ill patients further complicates causal attribution. Therefore, nephrotoxicity in this setting should be interpreted cautiously and may be considered a surrogate marker of clinical severity rather than a purely drug-related adverse event.

All patients in our series received combination therapy, reflecting the prevailing clinical strategy to enhance efficacy and mitigate resistance. Patients receiving multiple-antibiotic combinations experienced more severe clinical outcomes; however, this strategy did not translate into improved mortality or microbiological outcomes. Although more aggressive combinations are used in more severely ill children, their net advantage remains uncertain in terms of key outcomes [25].

From a clinical perspective, these findings underscore the importance of rational use of CST within antimicrobial stewardship frameworks. Given its nephrotoxic potential and limited impact in advanced critical illness, CST should be reserved for confirmed multidrug-resistant infections, with careful monitoring of renal function and avoidance of unnecessary concomitant nephrotoxic agents.

The logistic regression analysis in the present study revealed that patients without endotracheal intubation had the highest likelihood of microbiological eradication; in contrast, those with indicators of severe illness, such as intubation, PICU admission, or sepsis, were more likely to experience treatment failure. These findings are consistent with previous pediatric studies reporting that host-related factors, rather than the antimicrobial agent itself, play a decisive role in determining treatment outcomes in CST-treated infections. Studies conducted in PICUs have shown that the need for MV and prolonged PICU stay are strong predictors of mortality and poor microbiological response during CST therapy for MDR-GN infections [26, 27].

Our study also found that neutropenia significantly increased the risk of treatment failure, in line with earlier evidence suggesting that immunocompromised patients treated with CST experience higher mortality and lower microbiological eradication rates [28]. These findings emphasize that while CST retains potent in vitro activity against carbapenem-resistant organisms, clinical success is largely influenced by the patient’s immune competence, underlying comorbidities, and infection severity. Therefore, ensuring early diagnosis and managing critical illness-related complications may be just as crucial as antimicrobial selection in achieving favorable outcomes.

Another important consideration is the heterogeneity of infection types included in this cohort, encompassing bloodstream infections, ventilator-associated pneumonia, central nervous system infections, urinary tract infections, and other nosocomial infections. While this diversity reflects real-world clinical practice in tertiary pediatric centers, it may limit the ability to draw site-specific or pathogen-specific conclusions regarding CST efficacy.

Differences in tissue penetration, bacterial burden, host immune responses, and source control requirements across infection sites may have influenced treatment outcomes. In addition, the limited sample size within individual infection categories precluded robust subgroup analyses. Therefore, our findings should be interpreted primarily as reflecting overall clinical experience rather than as definitive evidence for specific infection syndromes.

This study has several limitations. First, its retrospective and single-center design limits the generalizability of the findings. Second, the sample size was relatively small, and some subgroups, particularly patients with specific infection types, contained only a few cases, which may have affected the statistical power of subgroup analyses. Third, systematic CST susceptibility testing by broth microdilution became available only after August 2023; therefore, earlier undetected resistance cannot be excluded, which may have influenced eradication rates.

This limitation may have influenced the observed microbiological eradication rates and could have led to an overestimation of treatment success in some cases. Therefore, our microbiological outcomes should be interpreted cautiously, particularly for infections treated before routine susceptibility testing was implemented.

As a result, the true prevalence of CST resistance might have been underestimated. Additionally, the heterogeneity in concomitant antimicrobial regimens and underlying comorbidities could have influenced treatment outcomes.

The strengths of this study include its conduct within a tertiary university hospital, which enables multidisciplinary patient follow-up and provides access to a large cohort of immunocompromised individuals. Furthermore, the Pediatric Infection Team's clinical management ensured comprehensive, specialized evaluation throughout the diagnostic and therapeutic processes. In addition, standardized data collection enhances the study's internal validity and strengthens the reliability of the findings. Collectively, these features demonstrate the methodological robustness of the research and underscore its potential to contribute meaningfully to the existing literature.

## Conclusion

CST remains a last-resort treatment for MDR and CR-GN infections in pediatric patients, particularly in settings with limited access to newer antimicrobial agents. In this study, despite relatively favorable microbiological eradication rates, outcomes were mainly influenced by infection severity and host-related factors rather than the antimicrobial regimen itself. Nephrotoxicity occurred in a minority of patients and was associated with increased mortality in univariable analyses. Notably, endotracheal intubation emerged as the strongest independent predictor of mortality**,** highlighting the critical impact of disease severity on treatment outcomes. Careful monitoring, rational antibiotic use, and improved access to novel antimicrobials are essential to optimize outcomes and preserve the effectiveness of CST.

## Supplementary Information

Below is the link to the electronic supplementary material.

Supplementary file1 (DOCX 14 KB)

## Data Availability

The datasets generated and/or analyzed during the current study are available from the corresponding author on reasonable request.

## References

[1] Tacconelli E, Carrara E, Savoldi A et al (2018) Discovery, research, and development of new antibiotics: the WHO priority list of antibiotic-resistant bacteria and tuberculosis. Lancet Infect Dis 18(3):318–327. 10.1016/S1473-3099(17)30753-3 29276051 10.1016/S1473-3099(17)30753-3

[2] van Duin D, Paterson DL (2016) Multidrug-resistant bacteria in the community: trends and lessons learned. Infect Dis Clin North Am 30(2):377–390. 10.1016/j.idc.2016.02.004 27208764 10.1016/j.idc.2016.02.004 PMC5314345

[3] Tamma PD, Aitken SL, Bonomo RA et al (2021) Infectious Diseases Society of America guidance on the treatment of extended-spectrum β-lactamase–producing Enterobacterales (ESBL-E), carbapenem-resistant Enterobacterales (CRE), and *Pseudomonas aeruginosa* with difficult-to-treat resistance (DTR-P. aeruginosa). Clin Infect Dis 72(7):1109–1116. 10.1093/cid/ciab295 33830222 10.1093/cid/ciab295

[4] Murray CJL, Ikuta KS, Sharara F et al (2022) Global burden of bacterial antimicrobial resistance in 2019: a systematic analysis. Lancet 399(10325):629–655. 10.1016/S0140-6736(21)02724-0 35065702 10.1016/S0140-6736(21)02724-0 PMC8841637

[5] Tamma PD, Han JH, Rock C et al (2015) Carbapenem therapy is associated with improved survival compared with piperacillin-tazobactam for patients with extended-spectrum β-lactamase bacteremia. Antimicrob Agents Chemother 59(9):5173–5179. 10.1128/AAC.02286-15 10.1093/cid/civ003 PMC4462658 25586681

[6] Kendrick JG, Carr RR, Ensom MHH (2017) Antibiotic dosing in obese children: a systematic review. Drugs 77(7):635–656. 10.1007/s40265-017-0851-9

[7] Sader HS, Mendes RE, Doyle TB et al (2021) Characterization of *Enterobacter cloacae* and *Citrobacter freundii* species complex isolates with decreased susceptibility to cephalosporins from United States hospitals and activity of ceftazidime/avibactam and comparator agents. JAC Antimicrob Resist 3(3):dlab136. 10.1093/jacamr/dlab136 34430873 10.1093/jacamr/dlab136 PMC8378278

[8] Buehrle DJ, Shields RK, Clancy CJ et al (2023) Carbapenem-resistant *Enterobacterales*: a review of epidemiology, treatment, and outcomes. Int J Antimicrob Agents 61(5):106744. 10.1016/j.ijantimicag.2023.106744 36738849 10.1016/j.ijantimicag.2023.106744

[9] Plachouras D, Karvanen M, Friberg LE et al (2009) Population pharmacokinetic analysis of colistin methanesulfonate and colistin after intravenous administration in critically ill patients with Gram-negative bacterial infections. Antimicrob Agents Chemother 53(8):3430–3436. 10.1128/AAC.01361-08 19433570 10.1128/AAC.01361-08 PMC2715599

[10] Rodríguez-Baño J, Gutiérrez-Gutiérrez B, Machuca I et al (2020) Treatment of infections caused by extended-spectrum-β-lactamase-, AmpC-, and carbapenemase-producing *Enterobacterales*. Emerg Microbes Infect 9(1):247–261. 10.1080/22221751.2020.1754133

[11] Bassetti M, Giacobbe DR, Aliberti S et al (2024) Management of severe infections due to carbapenem-resistant Gram-negative bacteria in critically ill patients. Crit Care 28:111. 10.1186/s13054-024-05031-w38581030

[12] Sahbudak Bal Z, Kamit Can F, Yazici P et al (2018) The evaluation of safety and efficacy of colistin use in pediatric intensive care units: results from two reference hospitals and review of literature. J Infect Chemother 24(5):370–375. 10.1016/j.jiac.2017.12.017 29361414 10.1016/j.jiac.2017.12.017

[13] Kidney Disease: Improving Global Outcomes (KDIGO) Acute Kidney Injury Work Group (2012) KDIGO clinical practice guideline for acute kidney injury. Kidney Int Suppl 2(1):1–138

[14] O’Grady NP, Alexander M, Burns LA et al (2011) Guidelines for the prevention of intravascular catheter-related infections. Clin Microbiol Infect 17(10):1254–1260. 10.1111/j.1469-0691.2011.03703.x21029248

[15] Kalil AC, Metersky ML, Klompas M et al (2016) Management of adults with hospital-acquired and ventilator-associated pneumonia: 2016 IDSA/ATS clinical practice guidelines. Clin Infect Dis 63(5):e61–e111 27418577 10.1093/cid/ciw353 PMC4981759

[16] Centers for Disease Control and Prevention. National Healthcare Safety Network (NHSN) patient safety component manual. U.S. Department of Health & Human Services; 2024. Available at: https://www.cdc.gov/nhsn/pdfs/pscmanual/pcsmanual_current.pdf.

[17] Centers for Disease Control and Prevention. National Healthcare Safety Network (NHSN) patient safety component manual: bloodstream infection (BSI) event. U.S. Department of Health & Human Services; 2024. Available at: https://www.cdc.gov/nhsn/pdfs/pscmanual/4psc_bsi_current.pdf.

[18] Goldstein B, Giroir B, Randolph A (2005) International pediatric sepsis consensus conference: definitions for sepsis and organ dysfunction in pediatrics. Pediatr Crit Care Med 6(1):2–8. 10.1097/01.PCC.0000149131.72248.E6 15636651 10.1097/01.PCC.0000149131.72248.E6

[19] van Duin D, Arias CA, Komarow L et al (2020) Molecular and clinical epidemiology of carbapenem-resistant *Enterobacterales* in the USA (CRACKLE-2): a prospective cohort study. Lancet Infect Dis 20(6):731–741. 10.1016/S1473-3099(19)30755-8 32151332 10.1016/S1473-3099(19)30755-8 PMC7473597

[20] Rodríguez-Baño J, Navarro MD, Retamar P et al (2012) β-Lactam/β-lactam inhibitor combinations for the treatment of bacteremia due to extended-spectrum β-lactamase–producing *Escherichia coli*: a post hoc analysis of prospective cohorts. Clin Microbiol Infect 18(6):563–571. 10.1111/j.1469-0691.2011.03570.x 10.1093/cid/cir790 22057701

[21] Kadri SS, Adjemian J, Lai YL et al (2018) Difficult-to-treat resistance in gram-negative bacteremia at 173 US hospitals: retrospective cohort analysis of prevalence, predictors, and outcome of resistance to all first-line agents. Clin Infect Dis 67(12):1803–1814. 10.1093/cid/ciy378 30052813 10.1093/cid/ciy378 PMC6260171

[22] Ozsurekci Y, Aykac K, Cengiz AB et al (2016) Is colistin effective in the treatment of infections caused by multidrug-resistant (MDR) or extremely drug-resistant (XDR) gram-negative microorganisms in children? Diagn Microbiol Infect Dis 85(2):233–238. 10.1016/j.diagmicrobio.2016.02.017 27041107 10.1016/j.diagmicrobio.2016.02.017

[23] Çetin ÇB, Özer Türk D, Şenol Ş et al (2016) Colistin efficacy in the treatment of multidrug-resistant and extremely drug-resistant gram-negative bacterial infections. Turk J Med Sci 46(5):1379–1384. 10.3906/sag-1506-125 27966301 10.3906/sag-1506-125

[24] Köksal E, Yılmaz E, Aydemir Ş et al (2017) Evaluation of colistin use in a pediatric intensive care unit. Turk J Med Sci 47(5):1523–1529. 10.3906/sag-1604-60

[25] Paterson DL, Ko W-C, von Gottberg A et al (2009) Antibiotic therapy for *Klebsiella** pneumoniae* bacteremia: implications of production of extended-spectrum β-lactamases. Clin Microbiol Infect 15(1):24–30. 10.1111/j.1469-0691.2008.02061.x 15206050 10.1086/420816

[26] Tamma PD, Turnbull AE, Harris AD et al (2013) Less is more: combination antibiotic therapy for the treatment of Gram-negative bacteremia in children. Pediatr Infect Dis J 32(12):1323–1328. 10.1097/INF.0000000000000117 10.1001/jamapediatrics.2013.196 PMC6857628 23921724

[27] Ustundag G, KaradagOncel E, Sahin A et al (2022) Colistin treatment for multidrug-resistant Gram-negative infections in children: caution required for nephrotoxicity. SiSli Etfal Hastanesi Tip Bulteni / The Medical Bulletin of Sisli Hospital 56(3):427–434. 10.14744/SEMB.2021.69851 10.14744/SEMB.2021.69851 PMC9580973 36304221

[28] KaradagOncel E, Ozsurekci Y, Kara A et al (2021) Evaluation of colistin use in pediatric intensive care units in Turkey. Medicine (Baltimore) 100(30):e44841. 10.1097/MD.0000000000044841

