# Supplementary material for: Real-world outcomes and safety of colistin therapy in children with multidrug-resistant gram-negative infections: a nine-year experience
Source: Eur J Pediatr. 2026 Mar 20;185(4):195. doi: 10.1007/s00431-026-06863-0 (PMC13002681; doi:10.1007/s00431-026-06863-0)
Supplement: Supplementary file 1 — Supplementary file1 (DOCX 14 KB) [file 431_2026_6863_MOESM1_ESM.docx]

Antibiotic susceptibility patterns of isolated organisms

|  | Susceptible | Intermediate | Resistant | Total |
| --- | --- | --- | --- | --- |
| Amikacin, n (%) | 31 (36) | 9 (10,5) | 46 (53,5) | 86 (100) |
| Gentamicin, n (%) | 19 (24,4) | 1 (1,3) | 58 (74,4) | 78 (100) |
| Ceftazidime, n (%) | - | 5 (9,3) | 49 (90,7) | 54 (100) |
| Cefepime, n (%) | - | 3 (4,8) | 59 (95,2) | 62 (100) |
| Ciprofloxacin, n (%) | 3 (3,8) | 11 (13,9) | 65 (82,3) | 79 (100) |
| Imipenem, n (%) | 1 (1,5) | 5 (7,5) | 61 (91) | 67 (100) |
| Meropenem, n (%) | 3 (3,5) | 9 (10,5) | 74 (86) | 86 (100) |
| Trimethoprim sulfamethoxazole, n (%) | 15 (24,2) | - | 47 (75,8) | 62 (100) |
| Colistin, n (%) | 5 (45,4) | 6 (54,5) | - | 11 (100) |
